# Design and Development of Liquid Drug Reservoirs for Microneedle Delivery of Poorly Soluble Drug Molecules

**DOI:** 10.3390/pharmaceutics11110605

**Published:** 2019-11-13

**Authors:** Mary-Carmel Kearney, Peter E. McKenna, Helen L. Quinn, Aaron J. Courtenay, Eneko Larrañeta, Ryan F. Donnelly

**Affiliations:** School of Pharmacy, Queen’s University, Belfast, 97 Lisburn Road, Belfast BT9 7BL, UK

**Keywords:** poorly soluble, hydrophobic, microneedles, transdermal, Nile red, atorvastatin, olanzapine, Franz cell

## Abstract

The poor aqueous solubility of existing and emerging drugs is a major issue faced by the pharmaceutical industry. Water-miscible organic solvents, termed co-solvents, can be used to enhance the solubility of poorly soluble substances. Typically, drugs with poor aqueous solubility and Log *P* > 3 are not amenable to delivery across the skin. This study investigated the use of co-solvents as reservoirs to be used in combination with hydrogel-forming microneedles to enhance the transdermal delivery of hydrophobic compounds, namely Nile red, olanzapine and atorvastatin. A custom-made Franz cell apparatus was fabricated to test the suitability of a liquid drug reservoir in combination with polymeric microneedles. A co-solvency approach to reservoir formulation proved effective, with 83.30% ± 9.38% of Nile red dye, dissolved in 1 mL poly(ethylene glycol) (PEG 400), permeating neonatal porcine skin over 24 h. PEG 400 and propylene glycol were found to be suitable reservoir media for olanzapine and atorvastatin, with approximately 50% of each drug delivered after 24 h. This work provides crucial proof-of-concept evidence that the manipulation of microneedle reservoir properties is an effective method to facilitate microneedle-mediated delivery of hydrophobic compounds.

## 1. Introduction

Poor drug solubility is a major challenge of many new and emerging therapeutic agents. It is frequently cited that 40% of recently marketed drug compounds have limited solubility in aqueous media [1], with this figure higher again for drugs in the developmental stage (>70%) [2]. This is problematic for several reasons, with one of the major issues being unacceptably poor drug bioavailability. As a result, many poorly soluble drugs are delivered using invasive hypodermic injections [3], or as high-dose oral preparations [4]. These approaches often result in manufacturing complexities and adverse effects, ultimately causing poorer treatment outcomes. To capitalise on the therapeutic potential of drugs that are promising in developmental stages but hampered by formulation issues due to their solubility profile, a variety of solubility enhancement strategies can be used [4]. These techniques are most commonly used for medications delivered orally but are not limited to this route of administration. 

Transdermal drug delivery is an attractive alternative route to oral and parenteral preparations. This non-invasive method of delivery offers many benefits including avoidance of first-pass metabolism, increased patient compliance and the ability to terminate drug delivery rapidly [5,6]. Unfortunately, however, it is limited in terms of the number of compounds that can permeate across the resilient barrier posed by the outermost layer of the skin, the *stratum corneum*. Various passive and active technologies have been developed that reduce the barrier effect of the *stratum corneum*, increasing the number of drugs amenable to transdermal delivery [7]. Microneedle (MN) technology is one of these that is rapidly advancing towards large-scale manufacture and commercialisation [8].

MNs are minimally invasive devices consisting of numerous micron-sized projections assembled on a baseplate. MNs penetrate the *stratum corneum* sufficiently to enable a port of access to the skin’s rich microcirculation yet are short enough to avoid painful stimulation of dermal nerves [5]. They can be subdivided into numerous different categories, each with their own distinct characteristics [9]. In this work, hydrogel-forming MNs have been used due to the vast number of advantages they confer [10]. When inserted into the skin, they are hard and sharp. Following insertion, they rapidly take-up interstitial fluid, swelling and forming a continuous hydrogel pathway through which drugs can diffuse [11]. To date, there have been relatively few reports of MNs being employed in transdermal delivery of hydrophobic molecules. This is most likely due to the inherent mechanism of action of MNs as they create aqueous channels within the skin. To facilitate MN-mediated delivery of poorly soluble drugs, alternative approaches to MN or MN-reservoir formulation need to be adopted. Ma and Gill (2014) demonstrated that they could coat solid MNs with a solid dispersion, whereby a poorly soluble compound (lidocaine) was dispersed within a hydrophilic matrix [12]. In vitro testing of this system demonstrated increased lidocaine delivery, in comparison with the marketed Emla^®^ cream. In a similar approach, Boehm et al. (2016) coated the poorly soluble antifungal agent, itraconazole onto poly(glycolic acid) MNs and demonstrated enhanced delivery [13]. Others have investigated dissolving MNs [14,15]. For example, dissolving MNs fabricated from oligomeric sodium hyaluronate and containing the poorly soluble and lipophilic compound artemether have been described [14]. Vora et al. (2018) have more recently used dissolving MNs for delivery of the hydrophobic compound, cholecalciferol [16]. While these examples provide considerable advancement to the transdermal delivery of poorly soluble compounds, both the coated and dissolving systems have limitations. Drug loading is limited to what can be coated onto the surface of the device with coated MNs, therefore, most amenable to highly potent molecules, rendering this a rather niche product. Although dissolving MNs have been developed to enable delivery of low potency substances at higher doses, delivery of hydrophobic drugs using dissolving MNs is typically limited to what can be loaded into the needles themselves, and so dosing capacity may again be limited. Coated and dissolving MNs have a part to play within advanced drug delivery, but alternative MN technologies such as hydrogel-forming MNs have been shown to overcome several of the previously described challenges [9]. Through the adoption of solubility enhancement strategies, there is potential to expand the MN drug delivery portfolio to include poorly soluble drugs.

For hydrogel-forming MNs to be used to deliver poorly soluble drug compounds, novel drug containing reservoirs displaying water miscibility as well as solubilising ability are required. Considering the various solubility enhancement techniques [2], a convenient and readily adaptable method is co-solvency. Co-solvency is a simple, popular approach used to enhance drug solubility. Drug substances are formulated in a liquid medium in which they display high solubility. Addition of a non-polar solvent reduces the polarity of the entire system, encouraging dissolution of hydrophobic molecules in aqueous media. It is important that the solvents chosen are biocompatible, non-toxic and relatively inexpensive [17].

This novel study presents proof of principle for the transdermal delivery of poorly soluble drugs using hydrogel-forming MNs, specifically focusing on the use of water-miscible, liquid co-solvents as drug reservoirs. As this is the first report of a liquid reservoir with hydrogel-forming MNs, development of an appropriate in vitro Franz cell setup is also described with Nile red (NR) dye (Log *P* = 3.8) used as a model hydrophobic compound in these studies. Delivery of the therapeutic compounds olanzapine (OLP) (Log *P* = 3.6), an antipsychotic compound and atorvastatin calcium trihydrate (ATR) (Log *P* = 5.4), a lipid regulating statin, were subsequently investigated using the developed in vitro method. 

## 2. Materials and Methods 

### 2.1. Materials

Poly(ethylene glycol) (PEG; MW 10,000, 600, 400 and 200 Da), methylene blue and HPLC grade methanol and acetonitrile were purchased from Sigma–Aldrich (Dorset, UK). ATR, NR, OLP and propylene glycol were purchased from Tokyo Chemical Industry UK Ltd. (Oxford, UK). Gantrez^®^ S-97, a copolymer of methyl vinyl ether and maleic acid (PMVE/MA, molecular mass: 1,500,000 Da) was a gift from Ashland (Kidderminster, UK). Anhydrous sodium carbonate was obtained from VWR International Ltd., (Poole, UK). Oxoid™ phosphate buffered saline (PBS) pH 7.4 tablets were obtained from Thermo Scientific (Sheffield, UK). Poly(ester) film, one-side siliconised, release liner, FL2000 PET 75 was purchased from Rexam Release B.V (Apeldoom, The Netherlands) Glisseal^®^N vacuum grease was purchased from Borer Chemie (Zuchwil, Switzerland). All other chemicals used were of analytical reagent grade.

### 2.2. Fabrication of Hydrogel-Forming Microneedle Arrays

Hydrogel-forming MNs were fabricated as described previously [11]. Briefly, polymer blends containing 20% *w*/*w* Gantrez^®^ S-97, 7.5% *w*/*w* PEG 10,000 and 3% *w*/*w* anhydrous sodium carbonate were cast into laser engineered silicone moulds. Cast moulds were centrifuged at 2205 g for 15 min using an Eppendorf^®^ 5804 series centrifuge (Eppendorf^®^ UK Ltd., Stevenage, UK) and the formulation dried under ambient conditions for 48 h. Crosslinking was induced by storing MNs at 80 °C for 24 h. Two array designs were fabricated, 11 × 11 (conical shaped; 600 µm height, 300 µm width at base and 150 µm interspacing) arrays and 19 × 19 (conical shaped; 600 μm height, 300 μm width at base and 50 μm interspacing). Mechanical properties of hydrogel-forming MN arrays were determined as previously reported [10]. 

### 2.3. Swelling Studies

The swelling of the hydrogel formulation was investigated using 1 cm^2^ needle-free baseplates, over 24 h at room temperature. Baseplates were prepared following the same method employed to fabricate MN arrays, except using silicone moulds containing no laser-engineered template for needle formation. The dry weight of each of these samples was recorded (*m_o_*). These were subsequently placed in disposable polystyrene weigh boats containing 30 mL of either phosphate buffered saline (PBS) (pH 7.4), glycerol, propylene glycol, PEG 600 or PEG 400. At regular intervals samples were removed from the solvents, blotted with tissue paper to remove excess solvent and weighed (*m_t_*). The same experiment protocol was employed, using ethanol as a co-solvent, with the weighing boats replaced by glass screw top jars to prevent ethanol evaporation. The percentage swelling was calculated using Equation (1):

(1) $$\%\;swelling=\left(\;\frac{m_{t}-m_{o}}{m_{o}}\right)\;\times \;100\;\%$$

### 2.4. Saturation Solubility

The saturation solubility of NR, OLP and ATR in various solvents was determined using the shake-flask method [18]. Briefly, an excess of drug was added to solvent in snap-top glass vials. Vials were sonicated for 10 min and placed in a rotary incubator (GFL Shaking Incubator 3033, Burgwedel, Germany) at 37 °C for 24 h at 40 rpm. Following incubation, samples were filtered through a 0.22 µm syringe filter (Chromacol, Herts, UK), diluted as appropriate and analysed using ultraviolet–visible (UV–vis) spectroscopy. Solvents tested included ethanol, PEG 200, PEG 400, PEG 600 and propylene glycol.

### 2.5. Development of In Vitro Liquid Delivery System

#### 2.5.1. Franz Cell Apparatus Modification

The conventional Franz cell setup, as described previously [19], is not suitable for testing in vitro delivery of a liquid formulation due to the presence of a steel weight which occludes the surface available for movement of liquid into, and through the MNs. This necessitated an alternative approach to be developed. Various modifications to conventional Franz diffusion cells (PermeGear Inc., Hellertown, PA, USA), were implemented to obtain a final set-up that provides a reliable and reproducible method of testing the in vitro permeation of liquids via hydrogel-forming MNs.

**Figure 1:**
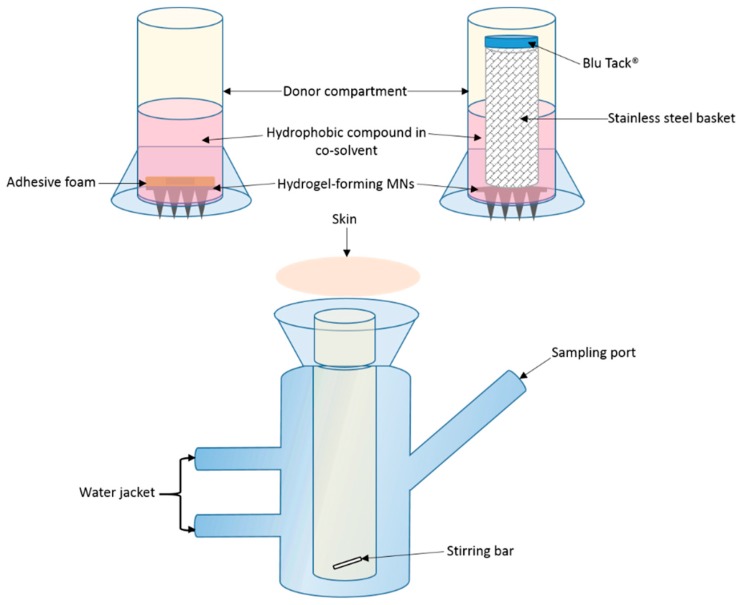
Schematic representation of modified Franz cell setup for in vitro drug permeation, with two donor compartment setups: foam adhesive and stainless steel basket with Blu Tack^®^ (not to scale).

Shaved dermatomed (Integra Life Sciences, Padgett Instruments, NJ, USA) neonatal porcine skin (thickness ~350 µm) was attached to the flat flange of a custom-made Franz cell donor compartment (15 mm orifice diameter, 40 mm height) using cyanoacrylate glue; *stratum corneum* side orientated upwards. The skin was placed on dental wax to provide support and to facilitate confirmation of MN penetration. MNs were inserted into the skin by applying gentle pressure to a syringe plunger for 30 s, with the flat end pressing on the baseplate. A stainless steel 40 Mesh Mini Basket (Quality Lab Accessories, LLC, Telford, PA, USA) was placed on top of the inserted MNs with Blu Tack^®^ (Bostik Ltd, Stafford, UK) attached to provide a mass of 8 g. Liquid drug formulation (1 mL NR dye in PEG 400, 50 µg/mL) was pipetted into the donor compartment, sealed using ParafilmM^®^ and mounted onto the receiving compartment. Receiving compartment contained 20% *v/v* PEG 400 in PBS (pH 7.4) stirred continuously at 600 rpm and thermostated at 37 ± 1 °C (Figure 1). Samples (200 µL) were removed at pre-defined time intervals and the receiver fluid replenished with an equivalent volume of degassed, pre-warmed receptor medium. Samples were centrifuged for 5 min at 12,225 g using an Eppendorf Minispin centrifuge (Eppendorf UK Ltd., Stevenage, UK) and then analysed using UV–vis spectroscopy. 

A variation of the above approach was adopted with the basket and Blu Tack^®^ replaced with an adhesive foam (prototype sample from TG Eakin, Comber, UK) with a central opening (outer diameter = 12 mm and inner diameter = 8 mm). The MN array was attached to the adhesive foam prior to insertion. The arrays were assembled with only the needle-free border of the array attached to the foam. All other aspects of the setup were the same as previously described, with the same sampling intervals and analytic methods adopted. 

#### 2.5.2. Effect of Stainless Steel Grid Design on In Vitro NR Permeation

**Figure 2:**
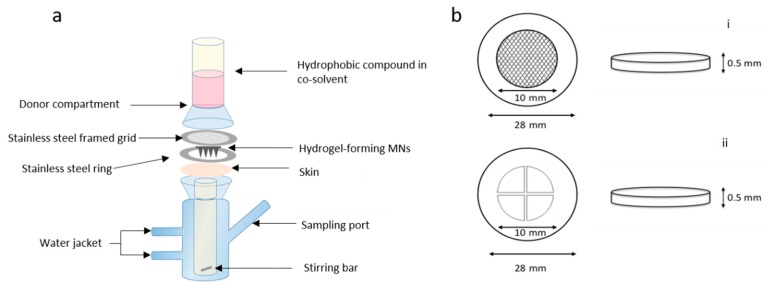
Schematic representation of (**a**) modified Franz cell setup for in vitro drug permeation with custom-made stainless steel ring and framed grid (not to scale) and (**b**) stainless steel disk (28 mm outer diameter, 10 mm inner diameter and 0.5 mm thick) with a (i) circular grid and (ii) cross-shaped grid.

Franz cell assembly was further modified to facilitate more efficient delivery of the drug from a liquid reservoir (Figure 2a). MNs were inserted into dermatomed neonatal porcine skin attached to a custom-designed stainless steel ring. To secure MNs in place, a stainless steel-framed grid (Currie Engineering, Coleraine, UK) was placed between a modified glass donor compartment and skin, which was secured in place with a clamp. Two grid designs were tested; both grids were laser cut into cylindrical stainless steel disks, 0.5 mm in thickness and an outer diameter of 28 mm (Figure 2b). The inner grid was either circular holes or cross shaped. The open area of the grid represented in Figure 2bi is 60 mm^2^ and 60.54 mm^2^ in Figure 2bii. All other aspects of the setup were the same as previously described above, with the same sampling intervals and analytical methods adopted.

#### 2.5.3. Effect of Microneedle Density on NR Permeation

MN arrays in an 11 × 11 configuration were initially used to create a pathway through the skin that enabled the permeation of NR from an organic solvent-based liquid formulation. Previous work completed by Garland et al. (2012) has investigated drug delivery from arrays containing 361 needles [20], approximately three times the number of needles in an 11 × 11 array. As such, it was hypothesised that by using 19 × 19 arrays, the permeation of NR could be increased by up to three-fold. MNs were tested using the same modified Franz cell set-up described previously with two designs tested, i.e., 11 × 11 arrays and 19 × 19 arrays. An aliquot (1 mL) of PEG 400 containing NR at a concentration of 50 µg/mL was filled into the Franz cell donor compartment. Samples (200 µL) were removed at regular intervals and replaced with fresh receiver media (PBS containing 20% *v/v* PEG 400). Samples were analysed using UV–vis spectroscopy. Following MN removal, skin samples were stained with methylene blue dye and visualised using a digital light microscope to assess MN insertion (Leica EZ4 D stereo microscope, Leica Microsystems, Milton Keynes, UK). Optical coherence tomography (OCT) (EX1301 OCT Microscope (Michelson Diagnostics Ltd., Kent, UK)) was used to visualise MN insertion of both array geometries, immediately post-insertion, in neonatal porcine skin, as previously described [21].

#### 2.5.4. Effect of NR Concentration on In Vitro Permeation

Two concentrations of NR dissolved in PEG 400 were tested. Aliquots (1 mL) of 50 µg/mL or 300 µg/mL NR in PEG 400 were filled into the donor compartment following assembly of the developed Franz cell apparatus. Receiver compartment composition was 20% *v/v* PEG 400 in PBS and 30% *v/v* PEG 400 in PBS for the 50 µg/mL and 300 µg/mL test solutions respectively. The remainder of the experimental set-up was as stated above. 

### 2.6. Investigation of OLP Stability 

OLP is recognised to display varying degrees of instability in both aqueous and biological matrices [22]. The extent of this instability is controversial, with contradictory reports on OLP behaviour in various environments [23,24,25]. As a result, OLP stability studies were conducted. The stability of OLP standards was assessed over 24 h to determine sample stability for analysis purposes. A further stability study of OLP reservoirs was conducted over 28 days to inform storage conditions of potential OLP reservoirs.

#### 2.6.1. OLP Stability over 24 h

The stability of OLP dissolved in a solution of 50:50 methanol:PBS was assessed across three concentrations. Working standards of 20 µg/mL, 10 µg/mL and 5 µg/mL were prepared by diluting the appropriate volume of a stock solution of olanzapine (0.5 mg/mL) with 50:50 methanol:PBS diluent solution. Solutions were stored at 37 °C for 24 h with 1 mL of the solution removed at pre-defined time intervals and analysed using reverse-phase high-performance liquid chromatography (RP-HPLC).

#### 2.6.2. OLP Stability in Organic Solvents over 28 days

The stability of OLP in three organic solvents, methanol, PEG 400 and propylene glycol, was assessed over 28 days. OLP solutions with three drug loadings were prepared (20 µg/mL, 10 µg/mL and 1 µg/mL) in each of the respective solvents. OLP solutions were divided into two cohorts with one group stored in light at ambient temperatures and the second group protected from light and stored in a refrigerator at a temperature between 2 and 8 °C. A sample was removed at pre-defined time intervals, filtered through a 0.22 µm syringe filter, and analysed using RP-HPLC. 

### 2.7. In Vitro Permeation of OLP and ATR

To determine whether hydrogel-forming MNs could be used to enhance the delivery of therapeutic compounds, liquid reservoirs containing either OLP or ATR were prepared. In vitro permeation was investigated using the custom-designed Franz cell set-up described previously. Following saturation solubility and swelling testing, PEG 400 and propylene glycol were selected as the most appropriate solvents to prepare both OLP-containing and ATR-containing liquid reservoirs. The saturation solubility of OLP in PEG 400 and propylene glycol was 31.59 mg/mL ± 3.15 mg/mL and 24.10 mg/mL ± 2.51 mg/mL, respectively. The saturation solubility of ATR in PEG 400 was 47.09 mg/mL ± 10.08 mg/mL and 48.43 mg/mL ± 4.55 mg/mL in propylene glycol. The receiver compartment contained PBS (pH 7.4) with 5% *v/v* of the solvent used in the drug formulation to maintain the solubility of the drugs in the receiver compartment and ensure sink conditions [26,27]. Sampling was conducted as described above for NR. For each organic solvent, two OLP concentrations were tested, i.e., 0.5 mg/mL and 2.5 mg/mL, and one ATR concentration was tested, i.e., 2 mg/mL. Samples were analysed using RP-HPLC.

### 2.8. Pharmaceutical Analysis

NR was quantified using UV–vis spectroscopy (PowerWave XS microplate spectrophotometer, BioTek^®^, Swindon, UK) with absorbance read at 570 nm. The saturation solubility analysis of OLP and ATR was determined using UV–vis spectroscopy, with absorbance read at 225 nm and 250 nm, respectively.

RP-HPLC (Agilent 1200^®^ Binary Pump, Agilent 1200^®^, Standard Autosampler, Agilent 1200^®^ Variable Wavelength Detector; Agilent Technologies UK Ltd., Stockport, UK) methods were developed and validated for both OLP and ATR. The chromatographic analysis of OLP in 50:50 methanol:PBS was achieved using a Waters™ XSelect CSH C18 column (75 mm × 3 mm internal diameter, 2.5 μm packing) with UV detection at 225 nm (run time of 5 min). Mobile phase consisted of 10:90 acetonitrile:0.025 M potassium dihydrogen phosphate buffer (pH 3.0) at a flow rate of 0.7 mL/min. The injection volume was 20 µL and column temperature was maintained at 20 °C. OLP samples were diluted in 50% *v/v* methanol in PBS (pH 7.4) prior to analysis, where necessary. The chromatograms obtained were analysed using Agilent ChemStation^®^ Software B.02.01.

The chromatographic analysis of ATR in PBS (pH 7.4) was completed as described previously [28]. Briefly, gradient separation was obtained using a Luna C18 (ODS1) column (150 mm × 4.6 mm i.d. with 5 µm packing; Phenomenex, Macclesfield, UK) with UV detection at 250 nm (run time of 20 minutes with post-run time set at 2 min). The mobile phase was a mixture of 25mM potassium dihydrogen phosphate buffer, pH 2.5, and methanol–acetonitrile (50:50, *v*/*v*), adjusted in composition over time. The injection volume was 20 µL and column temperature was maintained at 20 °C. 

The described methods for the quantification of OLP and ATR were validated according to guidelines from the International Conference on Harmonisation (ICH) [29]. Least-squares linear regression analysis and correlation analysis were performed on the triplicate calibration curves produced on each day of the validation period. This enabled the generation of the equation of each line and their respective coefficients of determination. To determine the individual limits of detection (LoD) and quantification (LoQ), an approach based on the standard deviation of the response and the slope of the representative calibration curve was employed.

### 2.9. Statistical Analysis

GraphPad Prism^®^ version 5.0 (GraphPad Software Inc., San Diego, CA, USA) was used to perform statistical analysis. Where appropriate, a Kruskal–Wallis test with post-hoc Dunn’s test was used for comparison of multiple groups, with a Mann–Whitney U test performed for comparison of two groups when *n* < 5. All data were expressed as the mean ± standard deviation. Statistical significance was denoted by *p* < 0.05 in all cases.

## 3. Results

### 3.1. Swelling Studies

**Figure 3:**
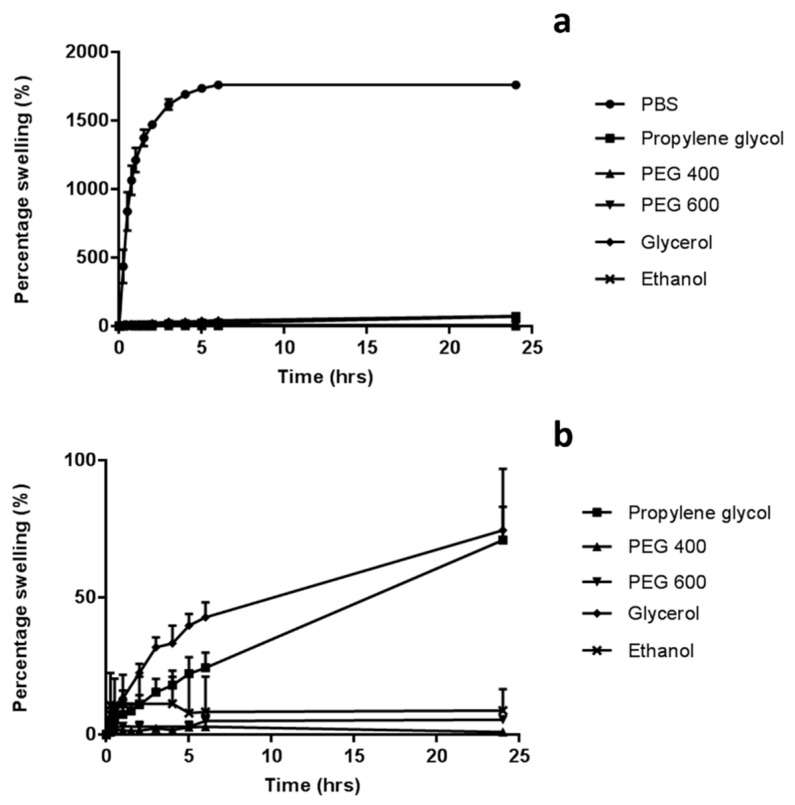
Percentage swelling of baseplates, cast from aqueous blends of 20% *w*/*w* PMVE/MA, 7.5% *w*/*w* poly(ethylene glycol) (PEG) 10,000 and 3% *w*/*w* Na_2_CO_3_, (**a**) in various organic solvents and phosphate buffered saline (PBS) and (**b**) in organic solvents only (Means ± S.D., *n* = 3).

The swelling profiles of hydrogel-forming baseplates in various solvents was investigated with findings presented in Figure 3. PBS (pH 7.4) was chosen as the standard swelling medium, as it is commonly used to represent interstitial fluid in in vitro experiments. In this medium, the percentage swelling of the hydrogel baseplates was 1760 ± 17.8%. In contrast, very limited, if any swelling occurred when baseplates were placed in organic media, with no significant difference found between the solvents (*p* > 0.05). There was virtually no swelling of the baseplate when immersed in PEG 400 for 24 h (<1%) and limited swelling with propylene glycol (70.9 ± 12.1%).

### 3.2. Development of In Vitro Liquid Delivery System

#### 3.2.1. Franz Cell Apparatus Modification

**Figure 4:**
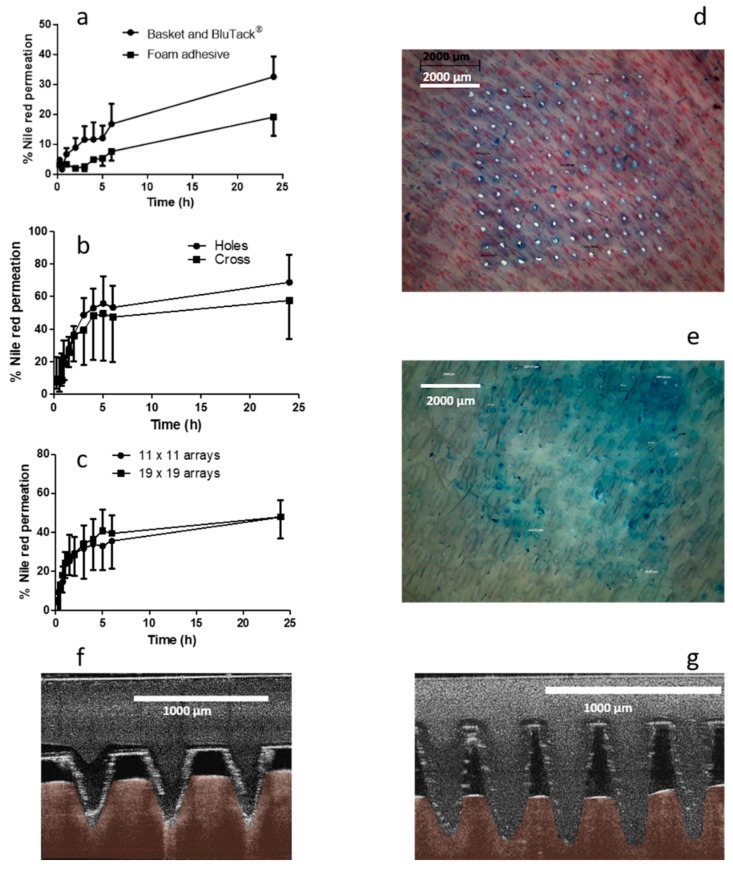
In vitro cumulative permeation profile of Nile red (NR) dissolved in PEG 400 across dermatomed neonatal skin using hydrogel-forming microneedle (MNs), (**a**) comparing two setups to maintain MNs in situ (means ± S.D., *n* ≥ 3), (**b**) comparing two stainless steel-grid setups to maintain MNs in situ (means ± S.D., *n* ≥ 3), (**c**) with either 11 *×* 11 or 19 *×* 19 dimensions (means ± S.D., *n* ≥ 3), digital images of methylene blue stained neonatal porcine skin following removal of (**d**) 11 *×* 11 and (**e**) 19 *×* 19 hydrogel-forming MNs, and optical coherence tomography (OCT) images of full thickness porcine skin immediately post insertion of (**f**) 11 *×* 11 (average MN insertion depth: 431.36 µm ± 7.9 µm) and (**g**) 19 *×* 19 (average MN insertion depth: 194.88 µm ± 13.81 µm) hydrogel-forming MNs.

Permeation of the model hydrophobic compound NR from a liquid reservoir required modification of the traditional Franz cell apparatus. Custom-designed donor compartments were fabricated with the capacity to hold up to 4 mL liquid. While the first set-up did facilitate NR permeation, as shown in Figure 4a, there were several replicates where no NR was detected in the receiver compartment. Apparatus was visually scrutinised upon dismantling to ascertain the source of error leading to lack of reproducibility. Skin visualisation images indicated that, in replicates where NR was detected in the receiver compartment, the MNs had penetrated and swollen while in place. Conversely, there were several replicates, where it was obvious that the MNs did not stay in place; there was a MN imprint, but it could be observed that the MNs had not swollen in the skin. To help promote insertion and retention of MNs in the skin, an alternative approach was adopted. Adhesive foam, with a central opening was prepared and used to retain the MNs in place, following insertion into the skin. Results presented in Figure 4a indicate that the drug was successfully delivered, however the rate and total percentage permeation was considerably, though not statistically (*p* = 0.267), reduced in comparison to that achieved in several instances with the basket and Blu Tack^®^ approach. Encouragingly, all replicates of the foam border approach resulted in NR permeation, however, the border of the foam reduced the surface area of the MNs exposed to the drug formulation, decreasing the rate of drug permeation.

#### 3.2.2. Effect of Stainless Steel Grid Design on In Vitro NR Permeation

Two grid designs were fabricated and tested with the aim of maintaining the swollen MNs in place. The total “open” area, i.e., the area through which liquid could flow, was similar for each grid. As is appreciable from the cumulative permeation profile shown in Figure 4b, there was no significant difference found between the two grid designs (*p* = 0.786). The final percentage permeation for the circular hole containing grid was 68.78% ± 16.84% and 57.65% ± 23.73% for the cross-shaped grid. Both in vitro setups provided reliable MN retention within the skin. Similar to previous approaches, skin was diligently examined following dismantling of the apparatus, with it observed that the MN arrays, in the majority of instances, were still fully inserted into the skin at 24 h. 

#### 3.2.3. Effect of Microneedle Density on NR Permeation

The effect of MN array density was investigated to determine the optimum array design for this application. Results from in vitro studies investigating NR are presented in Figure 4c. It is apparent, that in this instance, there is no significant difference between the two array densities with 47.78% ± 10.89% NR delivered after 24 h for 11 *×* 11 arrays and 47.93% ± 8.41% permeated after 24 h using 19 *×* 19 arrays (*p* = 1.00). Following dismantling of the apparatus, skin samples were stained with methylene blue dye and visualised using a light microscope. As shown in Figure 4d, 100% insertion was achieved using the 11 *×* 11 array design, however, insertion efficiency was considerably less for 19 *×* 19 arrays. OCT images (Figure 4e) also indicate that % insertion depth was greater for the 11 *×* 11 design in comparison to 19 *×* 19.

#### 3.2.4. Effect of NR Concentration on In Vitro Permeation

**Figure 5:**
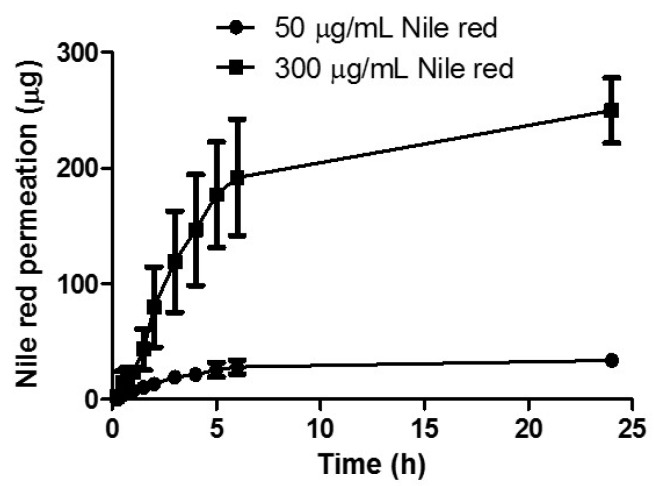
In vitro cumulative permeation profile of Nile red NR dissolved in PEG 400 across dermatomed neonatal skin using hydrogel-forming MNs from reservoirs containing 50 µg or 300 µg NR (means ± S.D., *n* = 3).

The effect of NR concentration on permeation was assessed by comparing the cumulative permeation profiles obtained following delivery from a reservoir containing 1 mL of 50 μg/mL NR in PEG 400 solution with a reservoir containing 1mL of 300 μg/mL NR in PEG 400 solution (Figure 5). After 24 h, 249.88 μg ± 28.15 μg was delivered from the 300 μg/mL reservoir, while 33.94 μg ± 3.00 μg was delivered from the lower loading reservoir. Increasing the loading by six times, resulted in a respective increase in drug permeation. As apparent from the cumulative percentage permeation profile, there was no significant difference between final NR delivery, with 83.30% ± 9.38% permeated from the higher loaded reservoir and 67.88% ± 6.01% permeated from the lower loaded reservoir (*p* = 0.20).

### 3.3. Investigation of OLP Stability 

#### 3.3.1. OLP Stability over 24 h

OLP stability over a 24 h period was assessed across three concentrations. The greatest reduction in OLP recovery was found in the lowest concentration solution (5 µg/mL) with a 11.11% ± 6.92% decrease in OLP present in the test solution after 24 h. The reduction in olanzapine was not as marked in the 20 µg/mL standard, with 94.81% ± 2.02% remaining after 24 h. Between 0 and 6 h, there was no more than 2% reduction across any of the standards, with a more notable decrease occurring between 6 and 24 h. 

#### 3.3.2. OLP Stability in Organic Solvents over 28 Days

**Figure 6:**
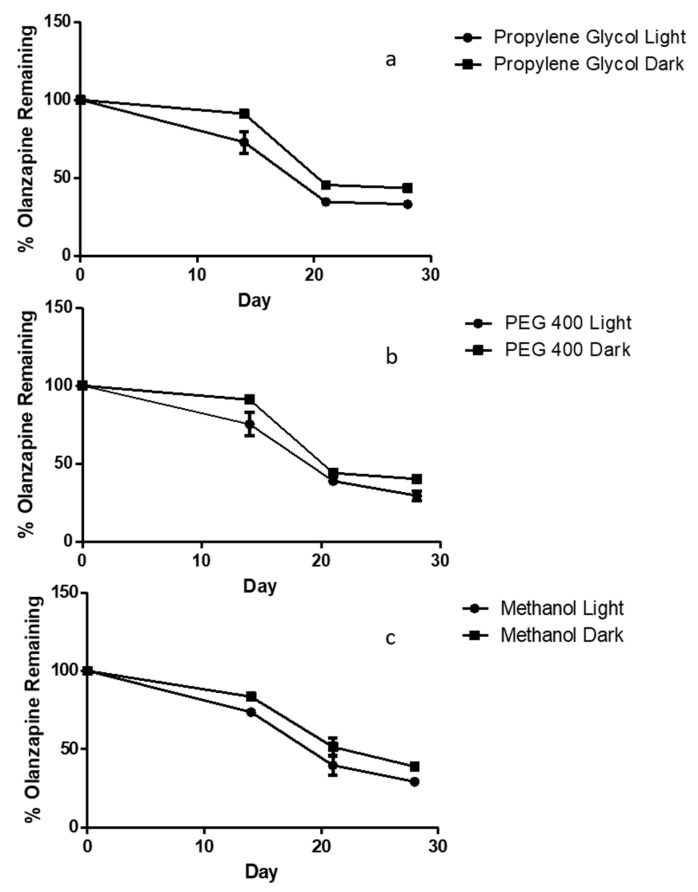
Stability profile of olanzapine over 28 days in solutions of (**a**) 5% *v/v* PEG 400 in PBS, (**b**) 5% *v/v* propylene glycol in PBS and (**c**) 5% methanol in PBS (means ± S.D., *n* = 3).

To determine OLP stability in potential liquid reservoirs, solutions of three concentrations were prepared in three different co-solvents. These were either stored at ambient temperatures, exposed to light or were protected from light and refrigerated between 2 and 8 °C. In all three co-solvents, those solutions protected from light degraded to a slightly lesser extent than those exposed to light. This difference is most relevant between day 0 and day 14, where protection from light and low temperatures maintained OLP concentrations above 90% of the original. In all co-solvents considerable OLP degradation occurred after 21 days. There was less than 50% of the original OLP remaining in all solvents exposed to light. The rate of degradation between day 21 and 28 slowed in comparison to day 14–21 (Figure 6).

### 3.4. In Vitro Permeation of OLP and ATR

**Figure 7:**
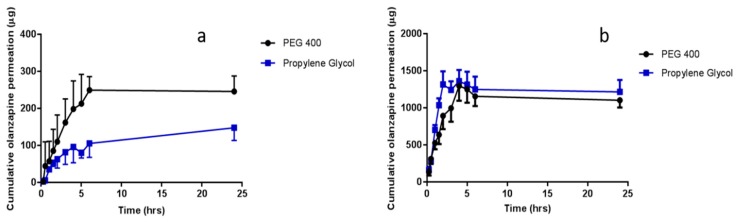
Cumulative in vitro olanzapine (OLP) permeation profile via hydrogel-forming MN arrays in combination with liquid reservoirs dissolved in PEG 400 or propylene glycol at an olanzapine concentration of (**a**) 0.5 mg/mL or (**b**) 2.5 mg/mL (means ± S.D., *n* ≥ 3).

**Figure 8:**
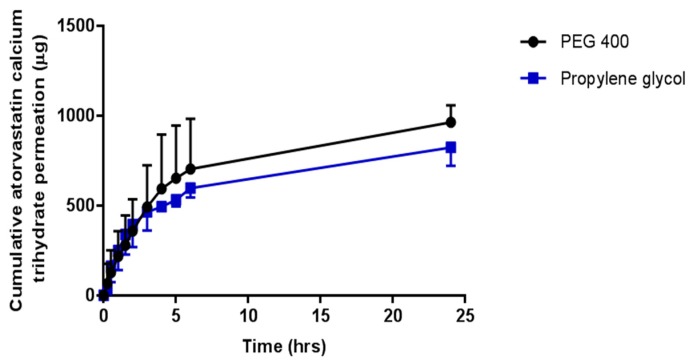
Cumulative in vitro atorvastatin calcium trihydrate (ATR) permeation profile via hydrogel-forming MN arrays in combination with liquid reservoirs containing 2.0 mg/mL atorvastatin calcium trihydrate dissolved in PEG 400 or propylene glycol (means ± S.D., *n* = 4).

**Figure 9:**
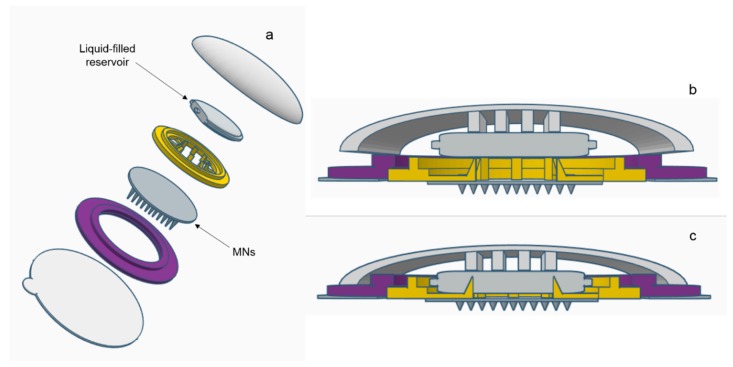
Schematic representation of a suitable device for the amalgamation of hydrogel-forming MNs and a liquid reservoir: (**a**) an expanded view of the device components and zoomed image of sealed liquid-containing reservoir, (**b**) the device on skin prior to the application of pressure and (**c**) the application of pressure with subsequent piercing of reservoir and insertion of MNs.

In vitro permeation studies of OLP and ATR from co-solvent drug reservoirs, across porcine skin, using hydrogel-forming MNs were conducted with results presented in Figure 7 and Figure 8, respectively. Test solutions were prepared at concentrations of either 0.5 mg/mL or 2.5 mg/mL for OLP studies and 2 mg/mL for ATR studies. Highest permeation from reservoirs containing 0.5 mg OLP was achieved using PEG 400 as the co-solvent, with 245.34 μg ± 41.74 μg permeated after 24 h. Interestingly, the highest permeation from 2.5 mg/mL OLP reservoirs was seen with propylene glycol. After 24 h, 1217.62 μg ± 221.60 μg OLP had permeated into the receiver compartment from the OLP in propylene glycol reservoir, with slightly less, but not statistically so (*p* = 1.00), permeated from the OLP in PEG 400 reservoir (1104.75 μg ± 221.60 μg). In terms of concentration effect, increasing OLP concentration by five times caused an almost directly proportional increase from reservoirs containing OLP in PEG 400. The most notable concentration dependent effect was observed with propylene glycol reservoirs, whereby a five times increase in OLP concentration resulted in eight times greater permeation. Considering the high solubility of ATR in PEG 400 and propylene glycol, these solvents were also tested for suitability as reservoirs for in vitro permeation of ATR across dermatomed neonatal porcine skin. The concentration of ATR in the receptor compartment of the diffusion cell increased progressively over the time period of the experiment. The cumulative mass of ATR delivered from both solvents across dermatomed (~350 μm) neonatal porcine skin over 24 h is displayed in Figure 9. There was no significant difference (*p* = 0.11) in the cumulative permeation of ATR from PEG 400 and propylene glycol after 24 h, with 962.6 ± 94.0 μg and 823.2 ± 102 μg delivered, respectively.

In terms of percentage permeation from reservoirs containing 0.5 mg OLP, 49.07% ± 8.35% of OLP in PEG 400 reservoirs was delivered after 24 h, with only 2.02% ± 1.15% from the control setup (no MNs inserted). When comparing this with the higher OLP loading using the same co-solvent system, there was no statistical difference (*p* = 0.397) with 44.19% ± 8.88% of total drug content delivered. This is also similar to ATR delivery using PEG 400 as the reservoir medium, with 48.13% ± 4.70% cumulatively delivered after 24 h. Percentage permeation of OLP increased from 29.50% ± 6.93% from reservoirs containing 0.5 mg OLP in propylene glycol to 48.70% ± 11.13% from 2.5 mg/mL OLP reservoirs. However, this increase was not significantly different (*p* = 0.114). By 24 h, 48.13% ± 4.70% of the drug content had been delivered from liquid reservoirs of ATR dissolved in PEG 400. The percentage drug permeation from the same volume and concentration of propylene glycol with hydrogel-forming MNs, under the same experimental conditions, was comparable to that achieved from PEG 400. For both PEG 400 and propylene glycol, no detectable ATR had permeated from control reservoirs (no MNs inserted) across the skin after 24 h.

## 4. Discussion

Water-miscible organics solvents, termed co-solvents, can be used to enhance the solubility of poorly soluble substances [30]. Relatively simple to use in a formulation and generally very effective, co-solvents are a popular pharmaceutical method used to increase solubility. Co-solvents in conventional transdermal systems often have a dual function; they act to increase drug solubility in the vehicle, whilst also acting as a permeation enhancer by increasing permeant partitioning into, and solubility within, the *stratum corneum*, thereby improving percutaneous absorption [31]. 

A range of water-miscible organic co-solvents were tested to determine those most appropriate for use with hydrogel-forming MNs in terms of hydrogel swelling. As expected, the highest degree of swelling was observed in 100% PBS. Contrastingly, when placed in a 100% organic solvent, the extent of swelling was significantly reduced for all solvents. This ensures that the MNs will not swell in the absence of physiological fluid. When considering an integrated MN patch consisting of hydrogel-forming MNs and a liquid reservoir, it would be imagined that the liquid reservoir would be maintained completely intact and separated from the array itself, yet contained within a singular patch, until the point of application. This is important to ensure the structural and mechanical integrity of the MN array itself as even a small degree of swelling may disturb the rigid hydrogel matrix in the dry state. Presented in Figure 9 is a schematic representation of a device that could be adapted to facilitate the combination of hydrogel-forming MNs and liquid reservoirs. Such a device would overcome the practical issue of liquid reservoirs such as those reported by Martanto et al. (2004) who reported the adhering of a ‘flanged glass chamber’ to the back of rats to retain liquid in place [32].

The saturation solubility of each test hydrophobic substance in various co-solvents was investigated to identify a suitable reservoir medium. The optimum solvent in terms of NR solubility was found to be PEG 400. However, OLP and ATR both displayed high solubility in PEG 400 and propylene glycol and so both co-solvents were further investigated. The concentration of each drug in the liquid reservoir in subsequent permeation studies was chosen to be notably lower than the experimental values obtained, to minimise the risk of drug precipitation. 

Co-solvents as drug reservoir media are beneficial in terms of drug solubility but can create challenges for experimental design. The Franz cell apparatus is the standard equipment used for in vitro testing of drug delivery via MN arrays [33,34,35,36]. To date, in vitro testing of hydrogel-forming MNs typically involves the use of a weight placed on top of the solid drug-containing reservoir to retain MNs in place. Liquid reservoirs have not been previously described in combination with hydrogel-forming MNs and, therefore, required a custom-designed donor compartment. This apparatus was designed to facilitate storage of a liquid reservoir, as well as preventing MN expulsion, while allowing movement of the liquid into the MN array. Following extensive preliminary work, an effective and reproducible experimental setup was established, as described above. Grids, of two different designs, were designed and fabricated to hold the MNs in place. Additionally, a stainless steel spacer ring of 0.5 mm thickness was used to facilitate MN swelling. Grid and spacer design was key to successful experimental set-up. 

Initial in vitro development studies investigated the effect of MN array density on drug permeation. It was assumed that if the number of individual MNs were increased by 3-fold, that there would be an equivalent 3-fold increase in NR permeation. However, this was not found to be the case. Microscope images obtained following removal of MNs indicated that MN insertion efficiency was superior for 11 × 11 arrays in comparison to 19 × 19. Post-experiment skin analysis showed differences in MN insertion efficiency. The design of the glass donor compartment prohibited manual thumb pressure being applied, which has previously been shown to be suitable for the insertion of 19 × 19 MN arrays into rats [37]. It has been shown that MN insertion depth is lower with 19 × 19 arrays when compared to 11 × 11 arrays, when an equivalent force is applied [38]. This may help explain the difference in the skin samples post MN removal. 

To establish the effect of drug loading on total permeation of NR, two donor solution concentrations were tested. As expected, increasing drug concentration caused a correlating increase in permeation, suggesting that concentration is a major influence on the transdermal delivery of NR using a liquid reservoir. Studying the profile in more detail, it is evident that there is no lag time with the linear period continuing for up to 5 hours. When MN arrays are inserted into the skin, they rapidly imbibe interstitial fluid. This fluid causes the MN array to swell, opening the hydrogel matrix and facilitating permeation of drug into the skin. As interstitial fluid is assimilated, it mixes with the liquid reservoir compartment and contributes to even more extensive swelling as the MNs swell from both upper and lower surfaces of the array. The rapid rate of hydrogel swelling, coupled with a liquid reservoir medium resulted in prompt and, and indeed rapid delivery of the hydrophobic compound. 

In vitro OLP permeation and ATR permeation from liquid reservoirs in combination with hydrogel-forming MNs was tested using the newly developed Franz cell model. At both a high (2.5 mg/reservoir) and low (0.5 mg/reservoir) OLP loading there was no significant difference between the two co-solvents in terms of cumulative drug permeation. It was taken into consideration that there was possible OLP degradation occurring in the receiver compartment between 6 and 24 h. Based on stability studies conducted it is known that OLP loss over this time duration is less than 10%. The slight reduction in OLP observed between these two points can, therefore, be accounted for. Results from ATR-containing reservoirs were similar to OLP with no difference found between propylene glycol and PEG 400. As the drugs were already in solution, the onset of permeation was rapid, with both drugs detected in their respective receiver compartments within 15 min of MN insertion. With respect to the drug not accounted for at 24 h, from a practical perspective, some of the liquid added to the donor compartment remained adhered to the glass sides of the vessel and on the surface of the skin. Some drug may also have been retained within the skin and the MN array itself. 

Comparing percentage permeation of OLP with ATR, using the same hydrogel-forming MNs, confirms co-solvency is a suitable method for facilitating MN-mediated delivery of hydrophobic drugs. If the permeant is soluble in the water-miscible solvent, diffusion through the hydrogel matrix can be facilitated. Solute-solvent interaction will also bear an effect on interaction of the solvent with the hydrogel matrix and, therefore, influence permeation, as demonstrated by the higher percentage permeation of NR. Further to this, as the MN system swells, the co-solvent is diluted and it is known from saturation solubility studies that increasing solvent polarity, readily reduces the solubility of the hydrophobic compound and could potentially cause precipitation of the drug within the hydrogel. This further highlights the importance of selecting a solvent in which the drug displays optimum solubility to minimise the likelihood of this occurring. The delivery of the drug in solution also increased the volume of co-solvent in the receiver compartment of the Franz diffusion cell, further aiding drug solubility in this medium. In vivo, it is thought that the rich dermal microcirculation may rapidly absorb the delivered drug, hastening its removal from the local area, thereby reducing the risk of drug precipitation and deposition in the predominantly aqueous environment of the viable skin. However, the exact drug permeation profile through the skin will have to be explored in greater detail.

Further improvements to solubility could be achieved through a combination of co-solvents and surfactants. However, the main challenge for delivery of the drug from a liquid reservoir in combination with MNs is appropriate device design. This work presented an adapted in vitro model which provided a robust method for assessing permeation. It would be envisaged that to progress this system, a suitable patch design would be required for in vivo application. An alternative approach could be to solidify the co-solvent system through the inclusion of higher molecular weight polymers, thereby having the solubilising enhancement effects of co-solvents but a more convenient formulation. 

Future work will include further investigation of the parameters governing drug permeation, considering in more depth the distribution profile of these drugs within the skin. Other hydrophobic compounds, as well as additional co-solvents will be investigated. Further to this, attempts will be made to solidify the system through the addition of higher molecular weight polymers, yet maintain the solubility enhancing effect provided by co-solvents.

## 5. Conclusions

Hydrogel-forming MNs are recognised as an advanced drug delivery system with multiple applications. Taking into account the increasing number of drugs displaying poor aqueous solubility, this work expands the realm of hydrogel-forming MNs beyond water-soluble compounds. There are numerous well documented advantages of transdermal drug delivery. However, if novel transdermal technologies do not cater for the largest proportion of emerging therapeutic compounds, the scope of MN application will be somewhat limited. The transdermal market, valued at approximately US $32 billion, is still based on only 20 drugs. Through the adoption of this technology, the number of drugs amenable to transdermal drug delivery could be significantly increased, expanding the market for current and emerging drugs. This presented work provides crucial proof-of-concept evidence that the manipulation of MN reservoir properties is imperative to the effective adaptation of hydrogel-forming MNs for hydrophobic compounds.
